# The importance of viral load in the severity of acute bronchiolitis in hospitalized infants

**DOI:** 10.6061/clinics/2021/e3192

**Published:** 2021-11-23

**Authors:** Milena De Paulis, Danielle Bruna Leal Oliveira, Luciano Matsumiya Thomazelli, Alexandre Archanjo Ferraro, Edison Luiz Durigon, Sandra E. Vieira

**Affiliations:** I Unidade de Urgencia e Emergencia Referenciada Infantil, Divisao de Pediatria, Hospital Universitario, Universidade de Sao Paulo, Sao Paulo, SP, BR.; II Unidade de Pronto Atendimento, Hospital Israelita Albert Einstein, Sao Paulo, SP, BR.; III Laboratorio de Virologia Clinica e Molecular, Departamento de Microbiologia, Instituto de Ciencias Biomedicas, Universidade de Sao Paulo, Sao Paulo, SP, BR.; IV Instituto Israelita de Ensino e Pesquisa Albert Einstein, Sao Paulo, SP, BR.; V Departamento de Pediatria, Faculdade de Medicina FMUSP, Universidade de Sao Paulo, Sao Paulo, SP, BR.; VI Plataforma Cientifica Pasteur, Universidade de Sao Paulo, Sao Paulo, SP, BR.

**Keywords:** Bronchiolitis, Rhinovirus, Respiratory Syncytial Virus, Human, Co-infection, Viral Load, Severity of Illness Index

## Abstract

**OBJECTIVE:**

The relationship between viral load and the clinical evolution of bronchiolitis is controversial. Therefore, we aimed to analyze viral loads in infants hospitalized for bronchiolitis.

**METHODS:**

We tested for the presence of human respiratory syncytial virus (HRSV) or human rhinovirus (HRV) using quantitative molecular tests of nasopharyngeal secretions and recorded severity outcomes.

**RESULTS:**

We included 70 infants [49 (70%) HRSV, 9 (13%) HRV and 12 (17%) HRSV+HRV]. There were no differences among the groups according to the outcomes analyzed individually. Clinical scores showed greater severity in the isolated HRSV infection group. A higher isolated HRSV viral load was associated with more prolonged ventilatory support, oxygen therapy, and hospitalization days, even after adjustment for the age and period of nasopharyngeal secretion collection. In the co-infection groups, there was a longer duration of oxygen therapy when the HRSV viral load was predominant. Isolated HRV infection and co-infection with a predominance of HRV were not associated with severity.

**CONCLUSION:**

Higher HRSV viral load in isolated infections and the predominance of HRSV in co-infections, independent of viral load, were associated with greater severity. These results contribute to the development of therapeutic and prophylactic approaches and a greater understanding of the pathophysiology of bronchiolitis.

## INTRODUCTION

Viral bronchiolitis (VB) is the most common infection of the lower airways in infants. Respiratory viruses are the principal causative agents, particularly human respiratory syncytial virus (HRSV) and human rhinovirus (HRV) (1). The rate of pediatric hospitalization due to VB is approximately 18% (2), wherein up to 20% them require intensive care unit (ICU) admission, and 2-3% need mechanical ventilation (3-5).

Several factors may be related to the severity of VB, including the causative agent, presence of comorbidities, and environmental factors. The literature is divided into the influence of viral load (VL) on the VB severity. Previous studies have shown that higher VL influences severity, especially HRSV (2,4,6-8). Nevertheless, there are differing opinions in the literature (9,10).

Therefore, this study aimed to analyze the association between VL and the clinical course of VB. This knowledge can improve prophylactic and therapeutic measures and aid the understanding of the pathogenetic mechanisms of VB in the future.

## METHODS

We included infants aged 0-6 months hospitalized from January 2013 to November 2015. The patients diagnosed with VB according to the American Academy of Pediatrics (11) criteria and nasopharyngeal aspirate samples (NPA) positive for HRSV or HRV as isolated agents or co-infected with both. We excluded those with recent (≤30 days before current infection) or current use of corticosteroids, current use of antibiotics (≤15 days), previous wheezing crisis, and congenital heart disease or bronchodysplasia.

NPA was collected during admission or within 72h of hospitalization. The automated extraction of the viral genetic material was performed using the Nuclisens^®^ Iso Kit (BioMerieux, Lyon, France) in the EasyMag equipment (BioMerieux, Lyon, France), and all protocols were processed as previously described (12). Quantification of VL was performed using quantitative polymerase chain reverse transcription (qPCR). In-house singleplex real-time (RT)-PCR assays for HRSV and HRV were performed using AgPath-ID™ One-Step RT-PCR reagents (Applied Biosystems) (13,14), and all extracts were tested for human RNase P (RNP) gene by RT-PCR to confirm sample quality. All extracts showed RNP cycle threshold (*Ct*) values (range, 18.3-31.4). We used a minimum of two dilutions of standard samples with pre-defined quantification, which allowed the absolute quantification of the number of copies/mL contained in each sample. The complete methodology is described in the Appendix.

The characteristics, background, and severity outcomes were collected from medical records and analyzed according to the etiological group. Demographic characteristics included age, sex, and race. The background included exclusive breastfeeding up to 6 months of life without introducing milk formula and exposure to smoking within the household. The severity outcomes were days of hospitalization and the need for and duration of ICU stay, non-invasive and mechanical ventilation, and oxygen therapy.

The infants were divided into the following groups: isolated HRSV infection, isolated HRV infection, and HRSV and HRV co-infection. The HRV co-infection was subdivided into the following subgroups: VL of HRSV greater than HRV and VL of HRSV less than HRV. The time elapsed from the onset of symptoms and collection of NPA was used to adjust the associations of higher HRSV VL and greater severity. Infants were also divided into groups based on the duration between the onset of symptoms and NPA collection (≤5 days and >5 days).

Demographic characteristics and background were analyzed using absolute and relative frequencies. The likelihood ratio test was used to calculate the associations with the etiological group. The severity outcomes were described using mean, standard deviation, median, minimum, and maximum and were compared using the Kruskal-Wallis test (Microsoft Excel 2003 software). The absolute VL was transformed to log_10_ copies/mL. A clinical score based on a latent variable was created (STATA 14, StataCorp. 2015. Stata Statistical Software: Release 14. College Station, TX: StataCorp LP). We started from the measured variables to create the latent variable: ICU stay (yes or no and the number of days), orotracheal intubation (yes or no and the number of days), CPAP (yes or no and the number of days), days of oxygen therapy, and hospitalization. The correlation between the binary variables and the respective number of days and between the ICU stay and the days of hospitalization was analyzed, resulting in a clinical score. The clinical score created was then categorized into tertiles; the first, second, and third tertiles were termed low-, medium-, and high- severity, respectively. Patients who stayed a maximum of 3 days in the hospital regardless of whether they used oxygen, but did not require ICU care were considered low-severity patients. Those who stayed for a maximum of 6 days in the hospital and required oxygen but did not require ICU care were considered medium-severity patients and those who stayed in the hospital for >6 days and required oxygen and ICU admission were considered high-severity patients.

In the univariate and multivariate analyses for the severity outcomes, adjustments were made for age (≤28 days, 28-90 days, ≥90 days) and the time between the onset of symptoms and collection of NPA (≤5 days, >5 days). Generalized linear models were built using a robust standard error estimate.

Fisher's test was used to analyze the distribution of VL categories according to severity categories. The level of significance was set at 5%.

For the sample calculation, assuming a correlation coefficient of 0.40 between VL and days of hospitalization and probability of type 1 error occurrence set at 5% and type 2 error occurrence set at 20%, 47 infants would be needed to find a significant correlation. The Research Ethics Committee approved the study of the University Hospital of the University of São Paulo (1664/17).

## RESULTS

**Figure 1:**
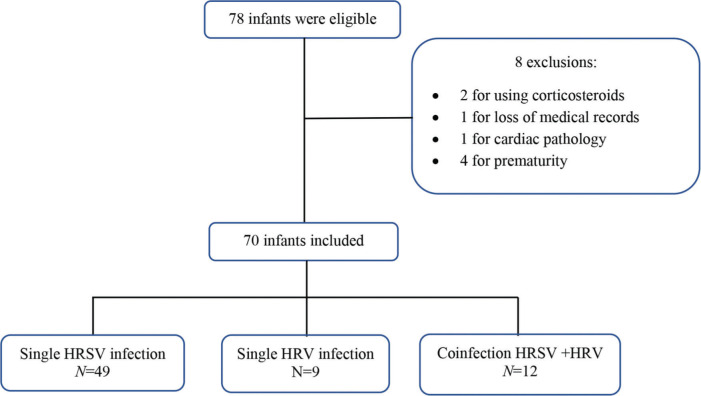
Characteristics of the patients included in the study. HRSV, human respiratory syncytial virus; HRV, human rhinovirus.

A convenience sample was selected, and 78 infants were eligible according to the inclusion criteria. After applying the exclusion criteria, 70 infants remained (Figure 1). Among these 70 infants, 49 had isolated HRSV infection, nine had isolated HRV infection, and 12 had HRSV+HRV co-infection.

The demographic and clinical characteristics were similar between the groups according to the etiology. The mean age was 70.4 days (standard deviation=46.8). Table 1 shows the descriptions and comparisons of infants according to their demographic and clinical characteristics according to etiology. Thirteen neonates were included (groups: HRSV, nine; HRV, two; HRSV+HRV, two). The average duration of hospitalization was 5 days. Two neonates were admitted to the ICU for ventilatory support.

Table 2 shows the comparative analyses of the severity outcomes according to the etiology.

According to the clinical score, isolated HRSV infection was associated with a higher number of high-severity cases than the other groups. A single HRV infection was associated with the least severe cases. In co-infections, when HRSV predominated over HRV, the cases were more severe (Table 3). The distribution of VLs according to severity categories and etiology is shown in Table S1-Appendix.

The median VL in isolated HRSV infection was higher than that in co-infections (6.6 *vs.* 5.5 log_10_ copies/mL, respectively; *p*=0.003). The median VLs of HRV in isolated infections and co-infections with HRV predominance were similar (4.5 *vs*. 4.4 log_10_ copies/mL, respectively; *p*=0.776).

Approximately 75% of the samples were collected within the first 5 days of symptom onset (mean, 4.5 days). We collected 25% of the samples after 5 days, with an average of 7 days from the onset of symptoms. The comparison of the median VL for isolated HRSV infection of infants who had the NPA collected with up to 5 days of symptom onset and those who had the NPA collected ≥5 days of symptom onset were similar (6.69 *vs.* 6.47 log_10_ copies/mL, respectively; *p*=0.462). This comparison showed similar results for infants with isolated HRV infection (5.04 *vs.* 3.98 log_10_ copies/mL, respectively; *p*=0.121). When comparing the medians of the VL of the HRSV, in the co-infections where HRV predominated, there was no difference concerning the day of NPA (6.37 *vs.* 5.27 log_10_ copies/mL, respectively; *p*=0.143). When HRV predominated, the medians for comparing HRV VL concerning the collection day were also similar (6.01 *vs*. 5·25 log_10_ copies/mL, respectively; *p*=1·000).

Table 4 shows the associations between the etiological groups and severity outcomes according to the univariate and multivariate analyses. The outcomes were adjusted for age (≤28 days; 29-90 days or >90 days) and time between symptom onset and NPA collection (≤5 days and >5 days). The coefficient represents the value of the average increase in days in the case of a higher VL.

## DISCUSSION

This study analyzed the importance of VL in bronchiolitis and showed that greater VL of HRSV was associated with greater severity in cases of infection by an isolated agent and co-infections.

The selected sample was representative of VB in infants, with a predominance of males and HRSV as causal agents. The clinical evolution was compatible with previous studies reporting an average of 5 days of hospitalization and approximately 20% of cases in the ICU (4,5).

In hospitalized infants with bronchiolitis, the most frequent etiological approach in clinical practice is qualitative analysis without VL quantification. According to each severity outcome, the analysis comparing etiological groups was insufficient to show any difference between these groups. In contrast, the use of the severity score made it possible to differentiate the etiological groups, highlighting HRSV as the agent associated with greater severity both in isolated infection and in co-infections in which it was the predominant agent.

The severity of infection can be influenced by the host characteristics, environment, and infectious agents. Confounding factors, such as the time elapsed between the onset of symptoms and NPA collection, could affect the analysis of clinical severity. Zhou et al. (7) and El Saleeby et al. (4) reported a decrease in the values of HRSV VL during hospitalization. Regardless of the drop in VL during hospitalization, the highest VL was associated with severity. In the present study, the associations of higher HRSV VL and greater severity remained significant even after adjusting for the time elapsed between the onset of symptoms and NPA collection. This result suggests a lack of bias in our study due to differences in VL according to the time when NPA was collected.

Notably, the association between severity and VL was not observed for HRV as an isolated agent, similar to previous studies (15,16). It is possible that the host's immune response and the HRV species involved in the infection are more critical than VL (16,17). The present study did not include an analysis of VL according to HRV species.

Respiratory virus co-infections are common in infants with lower airway infections (up to 40% of cases) (18). Other authors found greater severity in children with HRSV and HRV co-infection (6,19,20). However, in a systematic review, there was no influence on the severity outcomes (21).

Rodrigues et al. reported that the co-infection group had the predominance of HRSV, which was associated with greater severity (20). In addition to the results found in single HRSV infection, this suggests that the pathogenic mechanisms involved in HRSV bronchiolitis are associated with more severe clinical symptoms. One possible explanation is the competition between the pathogenic mechanisms of the viruses. HRSV is a slower-growing virus compared to HRV. However, when there is a delay in the onset of HRV infection, HRSV can block its replication, with a consequent decrease in HRV VL. It is also possible that interferon produced in HRSV infection causes the inhibition of HRV, decreasing the intensity of its manifestations (22).

The influence of age on the severity of bronchiolitis has been well described in the literature. The increase in VL in younger children, with consequently more severe clinical presentation, could be associated with indirect factors, such as exposure to infected contacts, viral serotype, and immaturity of the immune system. For example, Feikin et al. observed a tendency for VL to drop as age increased for HRSV (23). For HRV, there was a tendency to increase VL with older age. However, this study showed no difference in VL when considering age groups, both for isolated infections and for co-infection and severity, when multivariate analysis was carried out (23,24). This result could be explained by the inclusion of infants aged 0-6 months, the presence of maternal transplacental IgG, especially for HRSV, up to the third month of life, and the reduction of cytokine production by Th1 and Th2 cells in infants <4 months of age, conferring an anti-inflammatory protective effect and balancing the excessive inflammatory responses (25).

Regarding the influence of VL on the severity of bronchiolitis, other authors postulated that higher VL of HRSV is associated with less severe disease due to a more robust immune response, with faster viral clearing and decreased disease progression (10). On the other hand, some authors believe that greater VL corresponds with greater amplification of the immune response, a greater insult to the respiratory epithelium, and a greater degree of inflammation. As a result, the clinical presentation was more severe.

This study had limitations. First, the analysis of the immune response was not performed, which may contribute to clarify the influence of VL on clinical severity in future studies. Second, the lack of analysis of the various HRSV genotypes and HRV species and their possible relationships with VL and severity outcomes. The number of positive samples limited this analysis; therefore, new studies involving larger samples would be of significant relevance. Nevertheless, we used housekeeping gene quantification (human RNase P gene) to assure the quality of nasal lavage samples, although we did not correct VL by cell count of the samples.

## CONCLUSION

A higher VL of HRSV was associated with greater severity of isolated infections. In co-infections, the predominance of HRSV over HRV, independent of viral load, was associated with greater clinical severity. These results can contribute to the development of new therapeutic and prophylactic approaches and a deeper understanding of the pathophysiology of VB.

## AUTHOR CONTRIBUTIONS

De Paulis M and Vieira SE conceptualized and designed the study, drafted the initial manuscript, and reviewed the manuscript. Thomazelli LM, Oliveira DBL and Durigon EL were responsible for diagnostic laboratory analysis. Ferraro AA was responsible for the data analysis, and all of the authors approved the final version of the manuscript as submitted and agreed to be accountable for all aspects of the work.

## Figures and Tables

**Table 1:** Descriptive and comparative analyses of the demographic characteristics and background of the patients according to the etiology.

	Virus detected			Total (N=70)	*p*
	HRSV (N=49)	HRV (N=9)	HRSV+HRV (N=12)		
Sex, n (%)					0.806
Female	16 (32.7)	2 (22.2)	4 (33.3)	22 (31.4)	
Male	33 (67.3)	7 (77.8)	8 (66.7)	48 (68.6)	
Age (days)					0.864*
mean±SD	71.6±46.6	67.1±56.9	67.8±43.5	70.4±46.8	
median (min·; max·)	61 (11; 168)	51 (9; 167)	44·5 (24; 132)	54.5 (9; 168)	
Race, n (%)					0.238
White	32 (65.3)	5 (55.6)	5 (41.7)	42 (60)	
Mixed	14 (28.6)	4 (44.4)	7 (58.3)	25 (35.7)	
Black	3 (6.1)	0 (0)	0 (0)	3 (4.3)	
Breastfed**, n (%)^&^					0.570
No	22 (47.8)	2 (33.3)	4 (33.3)	28 (43.8)	
Yes	24 (52.2)	4 (66.7)	8 (66.7)	36 (56.3)	
Smoking***, n (%)^&^					0.170
No	19 (63.3)	4 (100)	6 (75)	29 (69)	
Yes	11 (36.7)	0 (0)	2 (25)	13 (31)	

Likelihood ratio test;

* Kruskal-Wallis test; & not all patients have the information.

** exclusive breastfeeding up to 6 months without introduction of milk formula.

*** Smoking: any smoker within the household.

HRSV, human respiratory syncytial virus; HRV, human rhinovirus.

**Table 2:** Severity outcomes according to the place of hospitalization, need and duration of ventilatory support and oxygen therapy and total hospitalization duration between the isolated HRSV, HRV, and HRSV co-infection with HRV groups.

	Virus isolated			Total (N=70)	*p*
	HRSV (N=49)	HRV (N=9)	HRSV+HRV (N=12)		
Hospital location, n (%)					0.625
ED**	11 (22.4)	5 (55.6)	3 (25)	19 (27.1)	
Ward	23 (46.9)	2 (22.2)	5 (41.7)	30 (42.9)	
Newborn nursery	5 (10.2)	1 (11.1)	1 (8.3)	7 (10)	
ICU	10 (20.4)	1 (11.1)	3 (25)	14 (20)	
ICU stay duration (days)					0.710*
mean±SD	1.55±3.8	0.56±1.67	2.42±5.81	1.57±4	
median (min.; max.)	0 (0; 15)	0 (0; 5)	0 (0; 20)	0 (0; 20)	
Ventilatory support, n (%)					0.631
No	42 (85.7)	8 (88.9)	9 (75)	59 (84.3)	
Yes	7 (14.3)	1 (11.1)	3 (25)	11 (15.7)	
CPAP, n (%)					0.286
No	43 (87.8)	9 (100)	10 (83.3)	62 (88.6)	
Yes	6 (12.2)	0 (0)	2 (16.7)	8 (11.4)	
BIPAP, n (%)					0.165
No	49 (100)	9 (100)	11 (91.7)	69 (98.6)	
Yes	0 (0)	0 (0)	1 (8.3)	1 (1.4)	
OTI, n (%)					0.701
No	45 (91.8)	8 (88.9)	10 (83.3)	63 (90)	
Yes	4 (8.2)	1 (11.1)	2 (16.7)	7 (10)	
Ventilatory support (days)					0.604*
mean±SD	0.98±3.04	0.33±1	1.42±2.64	0.97±2.78	
median (min.; max.)	0 (0; 14)	0 (0; 3)	0 (0; 7)	0 (0; 14)	
Duration of oxygen therapy (days)					0.171*
mean±SD	4.67±4.57	2.44±3.61	4.08±4.17	4.29±4.4	
median (min.; max.)	4 (0; 20)	1 (0; 10)	3 (0; 13)	3.5 (0; 20)	
Total hospitalization (days)					0.127*
mean±SD	5.8±5.4	2.8±3.9	6.4±6.7	5.5±5.5	
median (min.; max.)	4.7 (0; 25)	2 (0; 11.3)	4.3 (0; 23)	4.4 (0; 25)	

Likelihood ratio test;

* Kruskal-Wallis test.

** ED, emergency department: observation for >6 hours and <24 hours; ICU, intensive care unit; CPAP, continuous positive airway pressure; BIPAP, bilevel positive airway pressure; HRSV, human respiratory syncytial virus; HRV, human rhinovirus; OTI, orotracheal intubation; SD, standard deviation.

**Table 3:** Number and percentage of individuals in the groups according to the etiology and severity.

Etiology	Severity			
	Low	Moderate	High	Total
HRSV alone	12 (24.5)	19 (38.8)	18 (36.7)	49 (100)
HRSV > HRV	3 (33.3)	2 (22.2)	4 (44.4)	9 (100)
HRV > HRSV	2 (66.7)	0 (0.0)	1 (33.3)	3 (100)
HRV alone	7 (77.8)	0 (0.0)	2 (22.2)	9 (100)
Total	24 (34.3)	21 (30)	25 (35.7)	70 (100)

Fisher's chi squared test p=0.020.

HRSV, human respiratory syncytial virus; HRV, human rhinovirus.

**Table 4:** Univariate and multivariate analyses of the association between etiology and severity outcomes in bronchiolitis.

Etiology	Outcome	Crude Analysis				Adjusted Analysis*			
		Coefficient**	*p*	95% CI		Coefficient**	*p*	95% CI	
HRSV (n=49)	ICU stay (days)	1.91	0.050	0.00	3.82	2.03	0.036	0.14	3.93
	Resp. support (days)	1.78	0.018	0.30	3.25	1.84	0.021	0.28	3.39
	O_2_ therapy (days)	2.95	0.010	0.69	5.22	2.96	0.011	0.68	5.23
	Hospitalization duration (days)	3.23	0,014	0.65	5.81	3.23	0.012	0.70	5.75
HRV (n=9)	ICU stay (days)	1.25	0.276	-1.00	3.50	1.25	0.292	-1.07	3.57
	Resp. support (days)	0.75	0.276	-0.60	2.10	0.75	0.292	-0.64	2.14
	O_2_ threapy (days)	4.15	0.052	-0.03	8.33	2.22	0.328	-2.24	6.68
	Hospitalization duration (days)	4.06	0.093	-0.68	8.79	1.91	0.477	-3.35	7.16
HRSV/HR ratio (n=12)	ICU stay (days)	3.17	0.324	-3.13	9.47	3.59	0.270	-2.78	9.96
	Resp. support (days)	1.67	0.233	-1.07	4.40	1.91	0.167	-0.80	4.61
	O_2_ therapy (days)	4.17	0.042	0.15	8.19	4.78	0.017	0.86	8.69
	Hospitalization duration (days)	5.23	0.122	-1.40	11.85	6.33	0.060	-0.26	12.92

* Adjusted for age (≤28 days; 29-90 days or >90 days) and time between symptoms and collections (≤5 days or >5 days).

** average value of the increase (days) in case of having more viral load.

CI, confidence interval; HRSV, human respiratory syncytial virus alone; HRV, human rhinovirus alone; ratio, ratio of viral loads (HRSV/HRV) in co-infections. Viral load variables were dichotomized according to their median.

## APPENDIX

### Supplementary Material

**Total RNA/ and DNA extraction from nasopharyngeal aspirate samples**. For the extraction of the viral genetic material, 500 μL of sample from ANP was mixed with 500 μ of lysis buffer from the Nuclisens^®^ Iso Kit (BioMerieux, Lyon, France). In the same reaction, a negative (weather) and positive control (universal control gently provided by Dean Erdman/Atlanta). Briefly, 50 μL of magnetic silica was added to the sample/Lysis buffer mix, resulting in nucleic acid bonds. The nucleic acid-free components were removed in several washing steps performed on the Nuclisens EasyMag equipment (BioMerieux, Lyon, France). Finally, the nucleic acids were eluted from the solid phase with a Nuclisens Eluition Buffer (BioMerieux, Lyon, France) that keeps RNA intact for weeks at room temperature (23°C).

**Human respiratory syncytial virus and human rhinovirus detection using real-time quantitative polymerase chain reverse transcription**. Primers and probes previously described in the literature (1) were used (noninfluenza primer sets) kindly provided by the Centers for Disease Control and Prevention, Atlanta, GA, USA, for 14 different viruses: human respiratory syncytial virus (HRSV), human metapneumovirus, adenovirus, human rhinovirus (HRV), parainfluenza 1–4, coronavirus HKU1, NL63, 229E, and OC43; and commercial primers and probes for influenza A and B (Invitrogen). All the samples were amplified separately for the different viruses tested.

Real-time “one-step” amplification of HRSV and HRV was performed as described by Fry et al. (2) and Lu et al. (3) The amplification occurred in the Real Time 7300 PCR System thermocycler (Applied Biosystems) from the reverse transcription step (45°C/ 15′), followed by enzyme inactivation (94°C/5′), and finished with 45 cycles of 94°C for 15s for denaturing and 56°C for 30s for primer pairing. A minimum of two dilutions of standard samples with pre-defined quantification were used in RSV and RV. This allowed the absolute quantification of the number of copies/mL contained in each sample.

**Table S1:** Distribution of viral loads according to severity categories and etiology.

Etiology	Severity categories and viral load (Log_10_ copies/mL)								
	Low	95% CI HRSV	95% CI HRV	Medium	95% CI HRSV	95% CI HRV	High	95% CI HRSV	95% CI HRV
HRSV alone	6.46	5.99–6.87	-	6.36	6.07–6.85	-	6.94	6.55–7.20	-
HRSV>HRV*	6.67 >4.27	4.66–6.98	3.00–5.10	5.88>2.58	5.38–6.37	1.59–3.57	5.80>3.91	5.15–6.62	2.99–5.13
HRV> HRSV**	6.01 >3.16	1.70–4.62	5.01–7.00	0	0	0	5.25>3.44	3.44–3.44	5.25–5.25
HRV alone	4.33	-	1.13–6.52	0	0	0	5.20	-	4.64–5.75

* HRSV>HRV: human respiratory syncytial virus viral load predominates over human rhinovirus;

** HRV > HRSV: human rhinovirus viral load predominates over human respiratory syncytial virus; Cl, confidence
